# Preoperative diagnosis of a breast hydatid cyst using fine-needle aspiration cytology: a case report and review of the literature

**DOI:** 10.1186/1752-1947-6-293

**Published:** 2012-09-13

**Authors:** Maria Jesus Cancelo, Maria Martín, Nicolas Mendoza

**Affiliations:** 1 University Hospital of Guadalajara, University of Alcalá, Guadalajara, Spain; 2 Department of Obstetrics and Gynecology, University of Granada, Granada, Spain

## Abstract

**Introduction:**

A hydatid cyst of the breast is rare and often goes unnoticed by mammography and ultrasound. Preoperative diagnosis may be performed using fine-needle aspiration cytology, which also minimizes the risk of intraoperative rupture.

**Case presentation:**

We report the case of a 70-year-old Spanish woman who was diagnosed with a hydatid cyst using fine-needle aspiration cytology before surgery.

**Conclusion:**

Fine-needle aspiration cytology is an accurate and safe technique that can allow surgery to be avoided, especially in older patients or patients with high surgical risk.

## Introduction

Hydatid cyst disease is a zoonotic infection that results from tissue infestation with the larval stage of the parasite *Echinococcus granulosus*. The definitive hosts of the parasite are dogs, whereas the intermediate hosts are sheep and other ruminants. Humans are accidental intermediate hosts of this organism. It is an endemic disease that particularly affects people who live in rural areas in intimate contact with cattle. As in other countries of the Mediterranean basin, it is endemic in Spain, where it remains a serious health concern in many of the affected regions [1].

Hydatid cyst disease can affect all viscera and tissues of the body, with the liver and lungs being the most commonly involved; the kidneys, pancreas, bladder, spleen, ovary, brain, heart, thyroid, bone and muscle are rarely affected. Mammary tissue is also very rarely affected, accounting for only 0.27% of cases [2].

We present a case of an uncommon preoperative diagnosis following the fine-needle aspiration cytology (FNAC) of an isolated hydatid cyst of the breast in a 70-year-old woman, and a review of the literature.

## Case presentation

A 70-year-old Spanish woman was referred to our hospital for a mammographic diagnosis of a mammary nodule. She had no associated pain, nipple discharge, fever or family history of breast cancer. A clinical examination identified a lump in the upper inner quadrant of her left breast that was 2cm in size, nontender and freely mobile with regular borders. She had no axillary lymphadenopathy.

**Figure 1:**
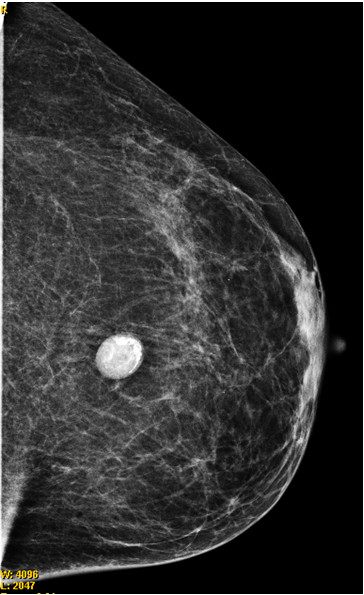
Mammogram scan showing a well-circumscribed, non-homogeneous nodule.

A mammographic scan showed a well-circumscribed area, 1.7cm in maximum diameter, with a calcified surface (Figure 1). A breast ultrasound showed a well-circumscribed area that was 1.7cm in size with both echolucent and echogenic areas. It had peripheral calcification. Doppler ultrasonography showed no vascularity in the lesion.

**Figure 2:**
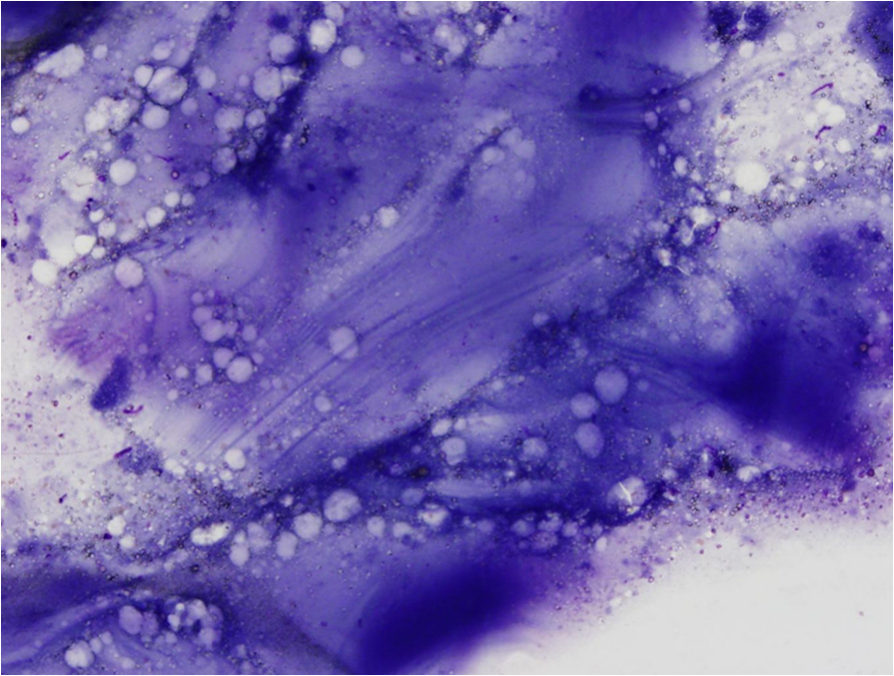
High-power view of the acellular membrane with striations (Diff-Quik stain, ×60).

FNAC of the lump revealed fragments of laminated membrane with parallel striations (Figure 2), dispersed retractile hooklets, granular debris, and isolated multinucleated giant cells. The diagnosis was consistent with a hydatid cyst.

An abdominal ultrasound, a chest X-ray and computed tomography scans of her abdomen and chest were normal. Hydatid serology was negative. An excision biopsy of the lump was planned, but our patient refused. Our patient was then monitored annually (for two years). Breast ultrasound and mammographic scans showed no changes.

## Discussion

Hydatid disease is an endemic illness in many parts of the world (the Mediterranean, South America, North and East Africa, Australasia, Russia and China) and, due to immigration and travel, its presence in non-endemic areas is possible [3]. It may affect almost any part of the body, occurring most frequently in the liver (75%), followed by the lungs (15%) and the remainder of the body (10%). The breast is very rarely the primary site of infection. Systemic dissemination is the most frequent source of breast hydatidosis. Patients typically present with a long history of a painless breast lump that slowly increases in size. The most frequent age of presentation is 30 to 50 years old, although ages from 20 to 74 years have been reported [4]. A summary of the management of hydatid cysts of the breast is presented in Table 1.

**Table 1:** Management of hydatid cysts of the breast

	
Clinical findings	→Mammary nodule
	→Other symptoms: pain, nipple discharge, fever
Differential diagnosis	→Cyst
	→Fibroadenoma
	→Phyllodes tumor
	→Circumscribed carcinoma
Diagnostic methods	→Mammography
	→Ultrasonography
	→Anti-echinococcus serum immunoglobulin E
	→Fine-needle aspiration cytology
Sonographic classification [5]	→Type 1: pure fluid collection
	→Type 2: fluid collection with a split wall
	→Type 3: fluid collection with septa
	→Type 4: heterogeneous echo patterns
	→Type 5: reflecting thick walls
	→“congealed water sign” [6]
Fine-needle aspiration cytology findings	→Hooklets, laminated membrane and scolices
Therapeutic options	→Cystectomy
	→Observation (in older patients or in high surgical risk)

Mammography and breast ultrasound scans are not very helpful for diagnosis because they do not provide specific findings, and hydatid cysts may be indistinguishable from benign cysts. Mammographic scans show a variety of imaging features that vary depending on the growth stage, associated complications and affected tissue. The characteristic ring-shaped structures inside the mass can be observed in over-penetrated scans. These structures are due to the differences in the densities of the walls or the contents of the daughter cysts within the main cyst. Magnetic resonance imaging may show a well-circumscribed cystic lesion with capsular enhancement, but this appearance resembles a breast abscess [4].

The sonographic appearance of mammary hydatid cysts may be similar to those observed in benign cysts, showing a well-defined, lobulated mass of heterogeneous echogenicity that may contain multiple cystic areas. However, the presence of a thicker and more laminated wall rather than a simple cyst and a thin layer of calcification within the lesion may suggest a hydatid cyst. Floating membranes, daughter cysts and vesicles are also revealed by ultrasonography. Gharbi *et al*. have described five types of ultrasound findings for hydatid cysts, including pure fluid collection (type I), fluid collection with a split wall (type II), fluid collection with septa (type III), heterogeneous echo patterns (type IV) and reflecting thick walls (Type 5) [5]. Durr-e-Sabih *et al*. have described another pattern called the ‘congealed water sign’, in which the hydatid fluid changes from a watery to a viscid gel. This change results in the appearance of curvilinear structures and intact folds of the germinal layer, which are trapped within the viscid matrix. They also noted that this sign was strongly suggestive of hydatid cysts [6]. Our case showed heterogeneous images on both mammography and ultrasonography, which were consistent with the type IV sonographic classification by Gharbi *et al.* (ultrasound not presented).

Specific immunological patterns are rarely used to diagnose hydatid cysts of the breast. Serologic tests, such as enzyme-linked immunosorbent assay, indirect hemagglutination and immunoblot techniques, can be used to confirm the hydatid origin of a cyst diagnosed by imaging techniques. Recently, the quantitative assessment of serum-specific immunoglobulin E by the ImmunoCAP® system has been performed for the diagnosis of hydatid cysts [7]. Elevated serum values of carbohydrate antigen 19.9 were found in the serum of a 69-year-old woman with a palpable mass in her breast, and these levels decreased after resection [8]. Diagnosis is frequently delayed because no specific signs are found at the time of examination, and they instead mimic other pathologies. A hydatid cyst is usually not included in the differential diagnosis of breast lumps due to its rarity, even in endemic areas. Due to the rarity of this condition, the above-mentioned mammographic and sonographic appearances of breast hydatid disease are frequently missed until an operative diagnosis has been made [9]. Rarely, a preoperative diagnosis can be made using a combination of clinical, imaging and FNAC findings. The presence of a laminated membrane with parallel striations, dispersed retractile hooklets, granular debris, and multinucleated giant cells is consistent with the diagnosis of a hydatid cyst. Only 12 reports have included cytological descriptions. No urticarial or anaphylactic reactions have been reported as a complication of this procedure. Therefore, FNAC can provide a safe, fast, inexpensive preoperative diagnosis and allow the planning of a cystectomy, minimizing the risk of intraoperative rupture [10].

The complete excision of the hydatid cyst without any spillage is the choice and definitive treatment of hydatid cyst disease, irrespective of its location. The aims of surgical treatment are the total removal of all parasitic elements, the avoidance of cyst content release and the maximum conservation of the affected viscera. Accidental implantation may be prevented by irrigation of the cyst bed with a 3% saline solution. One study has reported patients who underwent surgical cyst removal followed by medical treatment until the *Echinococcus* hemagglutination titers returned to normal [11].

Preoperative chemotherapy using albendazole has been shown to decrease the incidence of recurrent disease. However, it may not prevent disease recurrence in a distant site. Recurrence is typically due to either incomplete cyst removal or previously unidentified cysts. The reported recurrence rates range from 2% to 25% [12].

In our case, FNAC was harmless and was without complications or allergic reactions. After explaining the benign nature of the nodule, cyst excision was refused by our patient. The calcification and morphological stability of the nodule over time suggests an inactivate lesion.

## Conclusion

Hydatid disease of the breast is a very rare localization of this disease, but it should be considered as a differential diagnosis of breast lumps for individuals residing in endemic areas and among migrant populations.

The diagnosis is typically made postoperatively. Preoperative diagnosis is rare because the clinical and imaging findings are non-specific. FNAC can provide a safe, fast, inexpensive preoperative diagnosis and allow the planning of a cystectomy, minimizing the risk of intraoperative rupture.

## Consent

Written informed consent was obtained from the patient for publication of this case report and accompanying images. A copy of the written consent is available for review by the Editor-in-Chief of this journal.

## Competing interests

The authors declare that they have no competing interests.

## Authors’ contributions

MJC and MM analyzed and interpreted the patient data regarding the breast disease and the FNAC procedure. MJC and NM were major contributors in writing the manuscript. All authors read and approved the final manuscript.
